# Landscape of *TPMT* and *NUDT15* Pharmacogenetic Variation in a Cohort of Canadian Pediatric Inflammatory Bowel Disease Patients

**DOI:** 10.1093/ibd/izae109

**Published:** 2024-05-24

**Authors:** April M Kennedy, Anne M Griffiths, Aleixo M Muise, Thomas D Walters, Amanda Ricciuto, Hien Q Huynh, Eytan Wine, Kevan Jacobson, Sally Lawrence, Nicholas Carman, David R Mack, Jennifer C deBruyn, Anthony R Otley, Colette Deslandres, Wael El-Matary, Mary Zachos, Eric I Benchimol, Jeffrey Critch, Rilla Schneider, Eileen Crowley, Michael Li, Neil Warner, Dermot P B McGovern, Dalin Li, Talin Haritunians, Sarah Rudin, Iris Cohn

**Affiliations:** Division of Clinical Pharmacology and Toxicology, The Hospital for Sick Children, Toronto, Ontario, Canada; SickKids IBD Centre, Division of Gastroenterology, Hepatology & Nutrition, The Hospital for Sick Children, Toronto, Ontario, Canada; Child Health Evaluative Sciences, SickKids Research Institute, The Hospital for Sick Children, Toronto, Ontario, Canada; Department of Paediatrics, University of Toronto, Toronto, Ontario, Canada; SickKids IBD Centre, Division of Gastroenterology, Hepatology & Nutrition, The Hospital for Sick Children, Toronto, Ontario, Canada; Department of Paediatrics, University of Toronto, Toronto, Ontario, Canada; Department of Biochemistry, University of Toronto, Toronto, Ontario, Canada; Cell Biology Program, SickKids Research Institute, The Hospital for Sick Children, Toronto, Ontario, Canada; Institute of Medical Science, University of Toronto, Toronto, Ontario, Canada; SickKids IBD Centre, Division of Gastroenterology, Hepatology & Nutrition, The Hospital for Sick Children, Toronto, Ontario, Canada; Department of Paediatrics, University of Toronto, Toronto, Ontario, Canada; SickKids IBD Centre, Division of Gastroenterology, Hepatology & Nutrition, The Hospital for Sick Children, Toronto, Ontario, Canada; Child Health Evaluative Sciences, SickKids Research Institute, The Hospital for Sick Children, Toronto, Ontario, Canada; Department of Paediatrics, University of Toronto, Toronto, Ontario, Canada; Edmonton Pediatric IBD Clinic, Department of Pediatrics, University of Alberta, Edmonton, Alberta, Canada; Edmonton Pediatric IBD Clinic, Department of Pediatrics, University of Alberta, Edmonton, Alberta, Canada; Division of Pediatric Gastroenterology, Hepatology and Nutrition, Department of Pediatrics, BC Children’s Hospital, University of British Columbia, Vancouver, BC, Canada; Division of Pediatric Gastroenterology, Hepatology and Nutrition, Department of Pediatrics, BC Children’s Hospital, University of British Columbia, Vancouver, BC, Canada; SickKids IBD Centre, Division of Gastroenterology, Hepatology & Nutrition, The Hospital for Sick Children, Toronto, Ontario, Canada; CHEO IBD Centre, Division of Gastroenterology, Hepatology and Nutrition, Department of Pediatrics, Children’s Hospital of Eastern Ontario, CHEO Research Institute and Department of Pediatrics, University of Ottawa, Ottawa, Canada; Department of Pediatrics, Alberta Children’s Hospital Research Institute (ACHRI), University of Calgary, Calgary, Alberta, Canada; Division of Pediatric Gastroenterology & Nutrition, Department of Pediatrics, IWK Health Centre, Dalhousie University, Halifax, Nova Scotia, Canada; Division of Pediatric Gastroenterology, Hepatology and Nutrition, CHU Sainte-Justine, Montréal, Quebec, Canada; Section of Pediatric Gastroenterology, Winnipeg Children’s Hospital, University of Manitoba, Winnipeg, MB, Canada; Division of Gastroenterology, Hepatology and Nutrition, Department of Pediatrics, McMaster University, Hamilton, Ontario, Canada; SickKids IBD Centre, Division of Gastroenterology, Hepatology & Nutrition, The Hospital for Sick Children, Toronto, Ontario, Canada; Child Health Evaluative Sciences, SickKids Research Institute, The Hospital for Sick Children, Toronto, Ontario, Canada; Department of Paediatrics, University of Toronto, Toronto, Ontario, Canada; Institute of Health Policy, Management and Evaluation, University of Toronto, Toronto, Ontario, Canada; Faculty of Medicine, Memorial University, St John’s, Newfoundland & Labrador, Canada; Division of Gastroenterology and Nutrition, Department of Pediatrics, Montreal Children’s Hospital, Montreal, Quebec, Canada; Department of Pediatrics, Division of Pediatric Gastroenterology & Hepatology, Children’s Hospital Western Ontario, Western University, London, Ontario, Canada; The Centre for Computational Medicine, The Hospital for Sick Children, Toronto, Ontario, Canada; Cell Biology Program, SickKids Research Institute, The Hospital for Sick Children, Toronto, Ontario, Canada; SickKids IBD Centre, Division of Gastroenterology, Hepatology & Nutrition, The Hospital for Sick Children, Toronto, Ontario, Canada; F. Widjaja Foundation Inflammatory Bowel and Immunobiology Research Institute, Cedars-Sinai Medical Center, Los Angeles, California, USA; F. Widjaja Foundation Inflammatory Bowel and Immunobiology Research Institute, Cedars-Sinai Medical Center, Los Angeles, California, USA; F. Widjaja Foundation Inflammatory Bowel and Immunobiology Research Institute, Cedars-Sinai Medical Center, Los Angeles, California, USA; Division of Clinical Pharmacology and Toxicology, The Hospital for Sick Children, Toronto, Ontario, Canada; Division of Clinical Pharmacology and Toxicology, The Hospital for Sick Children, Toronto, Ontario, Canada; Department of Paediatrics, University of Toronto, Toronto, Ontario, Canada

**Keywords:** pharmacogenetics, thiopurines, genetic ancestry, *TPMT*, *NUDT15*

## Abstract

**Background:**

Patients with inflammatory bowel disease (IBD) exhibit considerable interindividual variability in medication response, highlighting the need for precision medicine approaches to optimize and tailor treatment. Pharmacogenetics (PGx) offers the ability to individualize dosing by examining genetic factors underlying the metabolism of medications such as thiopurines. Pharmacogenetic testing can identify individuals who may be at risk for thiopurine dose-dependent adverse reactions including myelosuppression. We aimed to evaluate PGx variation in genes supported by clinical guidelines that inform dosing of thiopurines and characterize differences in the distribution of actionable PGx variation among diverse ancestral groups.

**Methods:**

Pharmacogenetic variation in *TPMT* and *NUDT15* was captured by genome-wide genotyping of 1083 pediatric IBD patients from a diverse Canadian cohort. Genetic ancestry was inferred using principal component analysis. The proportion of PGx variation and associated metabolizer status phenotypes was compared across 5 genetic ancestral groups within the cohort (Admixed American, African, East Asian, European, and South Asian) and to prior global estimates from corresponding populations.

**Results:**

Collectively, 11% of the cohort was categorized as intermediate or poor metabolizers of thiopurines, which would warrant a significant dose reduction or selection of alternate therapy. Clinically actionable variation in *TPMT* was more prevalent in participants of European and Admixed American/Latino ancestry (8.7% and 7.5%, respectively), whereas variation in *NUDT15* was more prevalent in participants of East Asian and Admixed American/Latino ancestry (16% and 15% respectively).

**Conclusions:**

These findings demonstrate the considerable interpopulation variability in PGx variation underlying thiopurine metabolism, which should be factored into testing diverse patient populations.

**Key Messages**

**What is already known?**

Loss-of-function pharmacogenetic (PGx) variants in *TPMT* and *NUDT15* can increase the risk of thiopurine-induced myelosuppression and are differentially distributed among populations.

**What is new here?**

Approximately 11% of a large (*n* = 1083), diverse, Canadian pediatric IBD population was determined to be intermediate or poor metabolizers of thiopurines, with loss-of-function variants detected in *TPMT* across all genetic ancestries (Admixed American, African, East Asian, European, South Asian) and in *NUDT15* across all but African ancestry.

**How can this study help patient care?**

The interpopulation distribution of PGx variation in both *TPMT* and *NUDT15* highlights the need for healthcare providers to consider testing both genes to comprehensively assess patients’ risk of thiopurine-induced myelosuppression.

## Introduction

Inflammatory bowel disease (IBD), a disorder characterized by chronic inflammation of the gastrointestinal tract, requires ongoing medical management.^1,2^ Regions with high development indices such as Canada, the United States, and Europe contribute significantly to the prevalence of IBD, albeit the burden of disease is increasing in newly industrialized countries.^3^ Although much less often used in current practice in North America compared with the pre-biologic era, thiopurines, including azathioprine (AZA) and 6-mercaptopurine (6-MP), are still prescribed for pediatric IBD patients in some centers as monotherapy to maintain remission of Crohn’s disease (CD) or ulcerative colitis (UC), or in combination therapy with anti-tumor necrosis factor (anti-TNF) alpha agents to reduce the likelihood of formation of antidrug antibodies.^4^ Cost barriers and paucity of efficacy studies, particularly for the newer small molecule drugs, contribute to the ongoing use of immunomodulators in other healthcare systems.^5^ It is of utmost importance to optimize treatment approaches to improve patient outcomes and mitigate associated healthcare costs.

The efficacy of thiopurines in the treatment of pediatric CD and UC has been demonstrated in prior studies.^6–10^ However, the incidence of thiopurine-associated adverse events is well documented, affecting 20% to 40% of patients, and thus continues to be a significant concern in the management of IBD.^2,10–17^ Adverse effects of thiopurines include myelosuppression and hepatoxicity.^18^ Profound thiopurine-induced myelosuppression (TIM) due to altered drug metabolism is likely to occur early after commencement of treatment, but milder myelosuppression can develop at any point throughout the course of treatment. TIM is expected to impact approximately 7% of IBD patients. Myelosuppression can lead to consequential opportunistic infections and a mortality rate of 1% among patients with severe TIM.^14^

The thiopurine s-methyltransferase (TPMT) and nudix hydrolase 15 (NUDT15) enzymes play important roles in the metabolic pathway of thiopurines by regulating drug activation and restricting the formation and accumulation of toxic metabolites such as 6-thioguanine nucleotides (6-TGn) and thioguanine triphosphates. Deficiency in TPMT and NUDT15 enzyme activity can increase the risk of early profound TIM.^19–21^ Variation in the genes, *TPMT* and *NUDT15*, may lead to reduced enzyme activity in heterozygosity and enzyme deficiency in homozygosity. The distribution and frequency of *TPMT* and *NUDT15* alleles vary considerably between different ancestral groups. Approximately 10% of the global population is heterozygous for *TPMT* alleles that reduce enzyme activity and are considered intermediate metabolizers (IMs) of thiopurines. A further 0.33% of the population are TPMT-deficient such that thiopurine metabolism is shunted significantly towards 6-TGn.^22^ Reduced TPMT activity is predominately attributed to alleles *2, *3A, *3B, and *3C.^22,23^ The *3A allele is most common in European populations, whereas *3C is more prevalent in African and Asian populations.^24^ In general, individuals of European and African ancestry are typically more susceptible to TPMT-associated TIM.^25,26^ Conversely, variation in *NUDT15* tends to underlie TIM more commonly in individuals of Asian and Hispanic ancestry.^27,28^ The most common loss-of-function *NUDT15* allele, NUDT15*3, has been reported in approximately 6.7% and 6.1% of individuals of South Asian and East Asian ancestry, respectively.^29^ Deficiency of NUDT15 has been observed at a rate of approximately 1 in every 50 individuals of East Asian ancestry.^19^

Preemptive evaluation of TPMT and NUDT15 enzyme activity by pharmacogenetic (PGx) testing can identify patients who may be at an elevated risk for myelosuppression.^25,27,30,31^ It is widely recommended to assess TPMT enzyme activity prior to initiation of thiopurine therapy.^2,16,17^ Additionally, oversight groups such as the Clinical Pharmacogenetics Implementation Consortium (CPIC) and the Dutch Pharmacogenetics Working Group (DPWG) have published clinical guidelines detailing dosing recommendations based on *TPMT* and *NUDT15* genotypes.^19,32^ Genotyping recommendations are also detailed in drug monographs that have been approved by agencies such as the Federal Drug Administration (FDA), Health Canada-Santé Canada (HCSC), the European Medicines Agency (EMA), and Swissmedic.

To inform accurate pharmacotherapy guidance, it is imperative that PGx testing is reflective of variation in diverse populations. In this study, we analyzed genotype data from a large, ancestrally diverse cohort of Canadian pediatric IBD patients to assess the landscape of clinically actionable PGx variation in *TPMT* and *NUDT15*.

## Methods

### Study Cohort

The cohort included 1170 children and adolescents between 2 and 17 years of age enrolled in the Canadian Children Inflammatory Bowel Disease Network (CIDsCaNN) multi-center inception study at time of first diagnosis with IBD (CD, UC, or IBD-unclassified) at 1 of 12 participating sites nationally who had provided DNA for research. This ongoing cohort study is conducted in partnership with the CH.I.L.D. Foundation. Through participant and/or parental reports, the geographic ancestries of participants’ grandparents were captured at the time of enrolment via standardized forms. The self-reported ancestries were collapsed by researchers into 8 distinct ancestral groups (European, African, Caribbean/Latin/Central/South American, East/South East Asian, West Central Asian and Middle Eastern, South Asian, mixed, and other) using a modified Statistics Canada classification method.^33^ Additional cohort characteristics including the phenotypic spectrum have been previously described.^34^ The study protocol was approved by corresponding research ethics boards for each participating institution. Informed assent and consent were obtained from the participating children and their parents or legal guardians.

### Genotyping

DNA samples were collected from the 1170 participants for genotyping. Genotyping was performed at Cedars-Sinai Medical Center in a single batch using the Infinium Global Screening Array (GSA; Illumina Inc., San Diego, CA, USA). Samples that failed preliminary quality control criteria, including poor call rate, were excluded from downstream analyses. Samples were also excluded if the reported and detected biological sex was discrepant. After quality control, 1083 samples were included in downstream analyses of PGx variants. Imputation of missing genotype data was performed using the Michigan Imputation Server haplotype reference consortium panel.^35^ A post-imputation quality score of R^2^ >0.70 was applied to the data set.

### Genetic Ancestry Assignment

Principal component analysis (PCA) was performed to map participant DNA samples to genetic superpopulations from the 1000 Genomes Project (1KGP). The software tool, Peddy, was used to generate population clusters using a consistent subset of genetic loci that were trained on 2504 samples from diverse populations in the 1KGP data set.^36,37^ The first 3 principal components were plotted to assess population structure. Principal component analysis inferred populations were stratified and variants meeting Hardy Weinberg Equilibrium (HWE) *P* value >10^−6^ were retained for analysis. Genetic ancestry assignments were compared with self-reported ancestries to evaluate concordance.

### Pharmacogenetic Analyses

Analysis of PGx variation in *NUDT15* and *TPMT* was restricted to alleles that have been annotated by CPIC and the Pharmacogene Variation Consortium (PharmVar). The standardized PGx star (*) allele nomenclature system was used to assign haplotypes. A diplotype is the combination of the 2 haplotypes (star alleles) that an individual possesses. Diplotypes were automatically annotated using Stargazer v.2.0 (University of Washington), a software tool developed for calling PGx variation.^38^ Diplotypes were also determined by manual review of the genotype data for 10% of the cohort to assess concordance with Stargazer calls. Clinical PGx guidelines and drug labels were used to translate diplotype to phenotype (metabolizer status). The participant was assigned either normal, intermediate, poor, or indeterminate metabolizer status based on *TPMT* and *NUDT15* haplotypes. Normal metabolizers (NMs) exhibit typical enzyme activity and are considered to be wild-type (*1/*1), meaning that individual does not carry any known functional PGx variation that would otherwise impact enzyme activity. Individuals who are IMs have reduced enzyme activity, as they are heterozygous for a loss-of-function allele. On the other hand, poor metabolizers (PMs) have a deficiency in enzyme activity due to 2 copies of loss-of-function alleles. For phenotypic intermediate or poor metabolizers, a reduced starting dose or alternative therapy is indicated by CPIC, DPWG, and numerous drug monographs; thus, IM and PM status is considered clinically actionable. The fourth category, indeterminate metabolizers, includes individuals who are either heterozygous or homozygous for variants that have been previously annotated by PGx databases but have not yet been functionally characterized or with insufficient evidence to confidently assign function. The distribution of PGx diplotypes and the associated phenotypes was summarized for each genetic ancestral group. Fisher’s exact test was used to compare the proportion of metabolizer status phenotypes among the subpopulations within the cohort. Frequencies of clinically relevant, loss-of-function star alleles observed in each genetic ancestral group were compared with biogeographical population frequency estimates from CPIC using the χ2 test. The *TPMT* and *NUDT15* gene-specific information tables published by CPIC in 2018 were used to perform the statistical analyses.^24,29^ A *P* value of <0.05 was considered statistically significant.

## Results

### Ancestry Analyses

**Figure 1.**
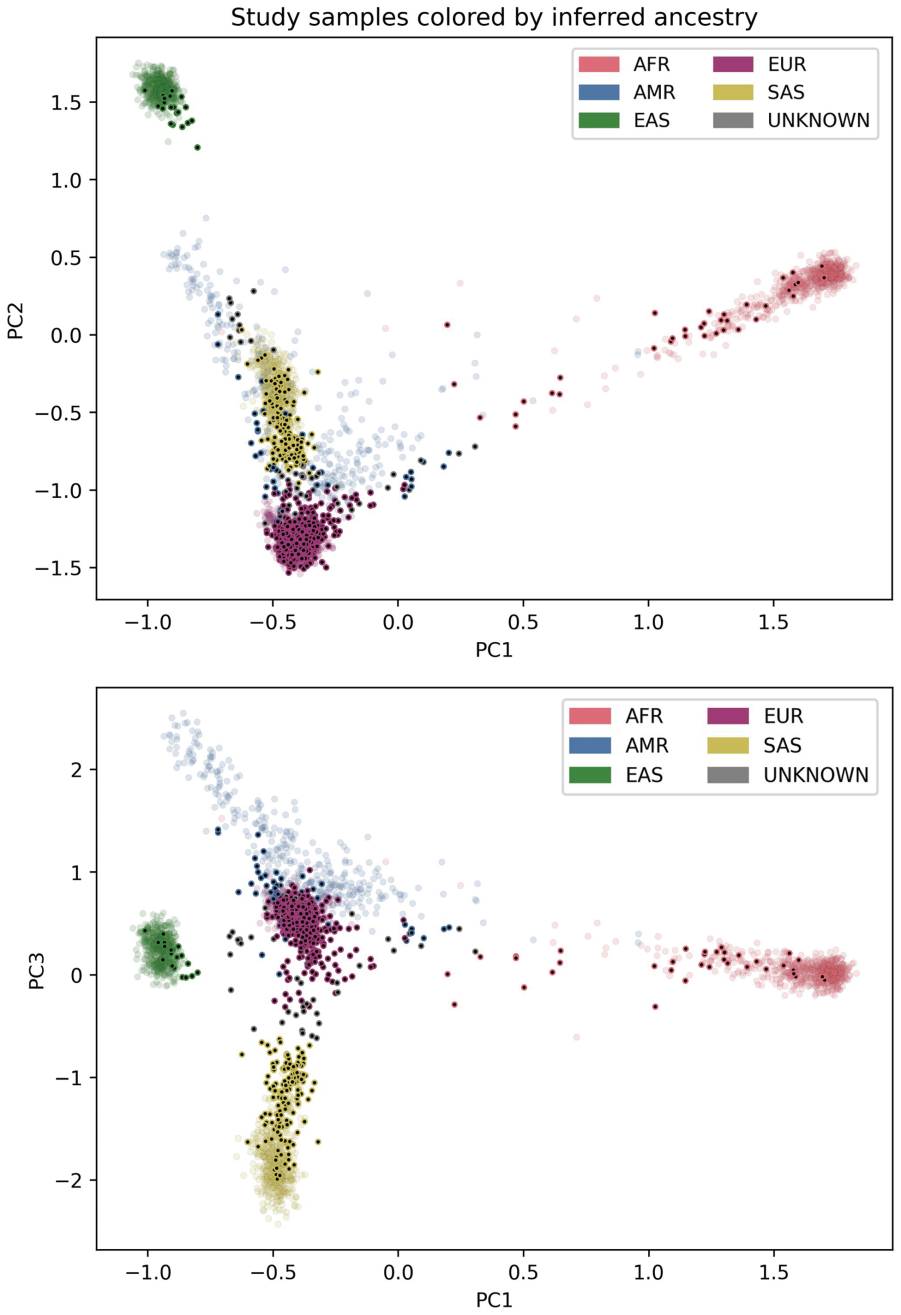
Principal component (PC) analysis plots (PC1 vs PC2; PC1 vs PC3) depicting inferred genetic ancestry of the CIDsCaNN cohort samples. The PC1 vs PC3 plot exhibits distinct sample clustering with respect to 5 superpopulations from the 1KGP database. Abbreviations: AFR,  African; AMR,  Admixed American; EAS,  East Asian; EUR,  European; SAS, South Asian.

The cohort samples mapped onto 5 distinct 1KGP superpopulations using the first 3 principal components. The clusters represent Admixed American, African, East Asian, European, and South Asian genetic ancestry (Table 1). Most samples mapped to European ancestry (*n* = 794), followed by South Asian (*n* = 150), Admixed American (*n* = 40), African (*n* = 36), then East Asian (*n* = 19; Figure 1). Genetic ancestry could not be resolved for 44 individuals. Of these 44 participants, 35 reported their ethnicity, with 77% (*n* = 27) having reported mixed ethnicity or ethnicity that is not reflected in the 1KGP data set. An additional 881 participants shared their ethnic background, of which 754 selected a singular ethnic background that is reflected in the 1KGP data set. For this subset of the cohort, 97% concordance between genetic ancestry and self-identified ethnic background was observed. For subsequent analyses, genetic ancestry was used to evaluate distribution of PGx variation among populations.

**Table 1.** Description of ancestries from the phase 3 release of the 1000 Genomes Project (*n* = 2504).

1KGP Superpopulation	Subpopulations
AFR (African)	Esan in Nigeria Gambian in Western Division, Mandinka Luhya in Webuye, Kenya Mende in Sierra Leone Yoruba in Ibadan, Nigeria African Caribbean in Barbados African Ancestry in Southwest USA
AMR (Admixed American)	Colombians in Medellin, Colombia Mexican ancestry in Los Angeles, CA, USA Peruvians in Lima, Peru Puerto Ricans in Puerto Rico
EAS (East Asian)	Chinese Dai in Xishuangbanna, China Han Chinese in Beijing, China Southern Han Chinese Japanese in Tokyo, Japan Kinh in Ho Chi Minh City, Vietnam
EUR (European)	Utah residents with Northern and Western European ancestry British in England and Scotland Finnish in Finland Iberian Populations in Spain Toscani in Italia
SAS (South Asian)	Bengali in Bangladesh Gujarati Indians in Houston, Texas, USA Indian Telegu in the UK Punjabi in Lahore, Pakistan Sri Lankan Tamil in the UK

1KGP: 1000 Genomes Project.

Adapted from the 1000 Genomes Project Consortium, 2015 (37).

### *TPMT* Genotyping

After imputation and quality control, 5 *TPMT* star alleles designated as loss-of-function by CPIC and PharmVar were available for evaluation (*2, *3A, *3B, *3C, *41; Supplementary Table 1). These alleles were captured by the array and did not require imputation. Six additional characterized loss-of-function alleles (*4, *11, *14, *15, *23, *29) that are rare in the general population were not imputed with sufficient quality and were thus excluded from analyses. Alleles *3B and *41 were not detected in the cohort, which is expected as *TPMT* deficiency has been predominately attributed to alleles *2, *3A, and *3C.^19^ Participants determined to be IMs of thiopurines thus had 1 of 3 possible *TPMT* diplotypes (TPMT*1/*2, *1/*3A, or TPMT*1/*3C). The only *TPMT* diplotype corresponding to a PM phenotype detected in the cohort was *3A/*3A.

**Figure 2.**
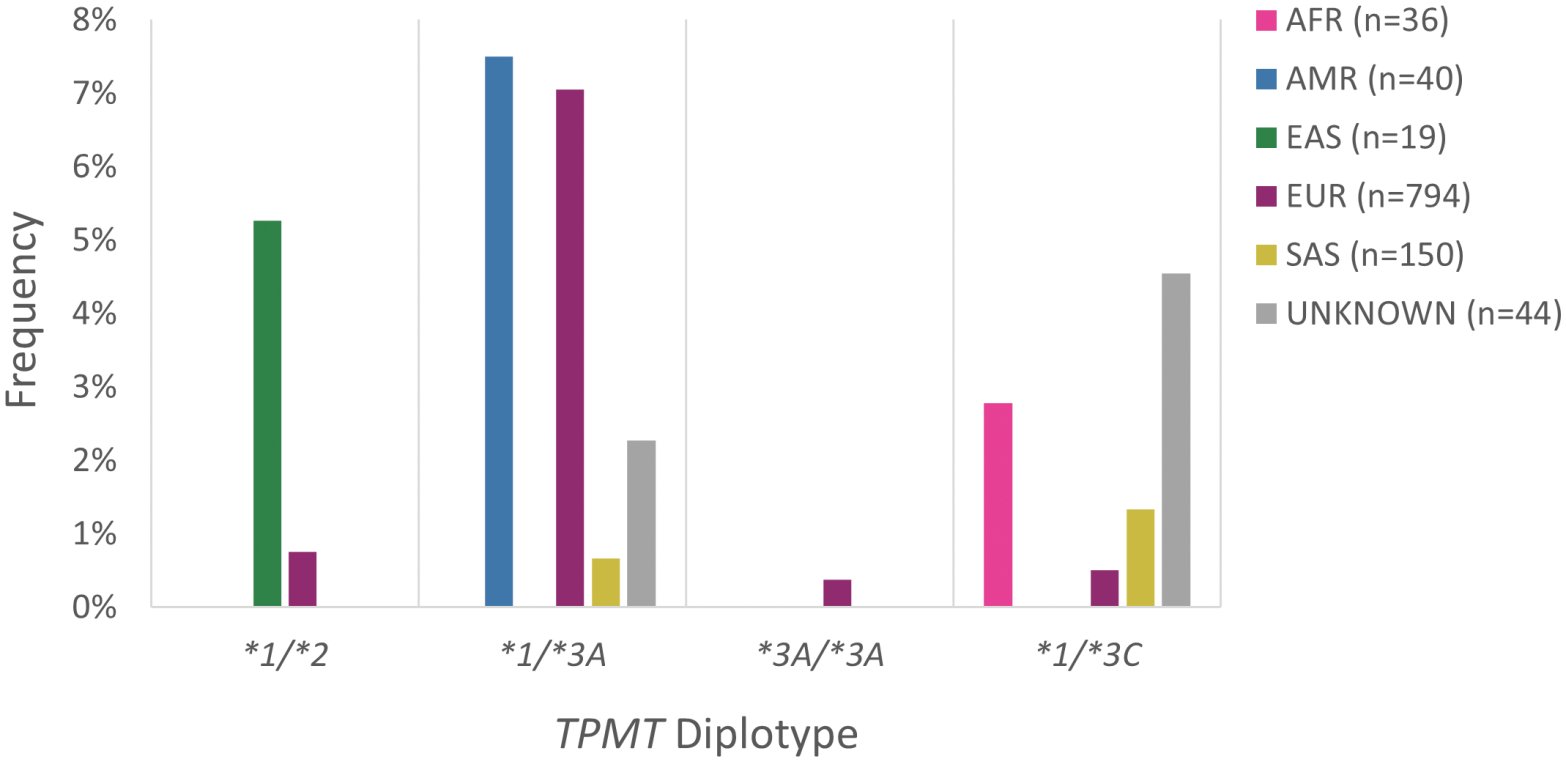
Frequency of *TPMT* diplotypes associated with a non-standard thiopurine dosing recommendation as per published PGx guidelines and drug label annotations, stratified by genetic ancestral group. Diplotypes *1/*2, *1/*3A, and *1/*3C are associated with intermediate metabolizer status. The *3A/*3A diplotype is associated with poor metabolizer status. Abbreviations: AFR, African; AMR, Admixed American; EAS, East Asian; EUR, European; SAS, South Asian. The “unknown” ancestral group corresponds to samples whereby ancestry could not be resolved by principal component analysis due to genetic admixture.

The *1/*2 diplotype was detected in individuals of European and East Asian Ancestry at frequencies of 0.76% and 5.3%, respectively (Figure 2). TPMT*2 is globally rare and has predominately been reported in individuals of European, African, and Admixed American ancestry.^24^ In these 3 populations, the frequency of the *2 allele was consistent with estimates provided by CPIC for similar biogeographical groups (Supplementary Figure 1). The frequency of TPMT*2 in participants of East Asian ancestry differed significantly (*P* < .001) from the corresponding CPIC estimate. The frequency reported herein may appear inflated due to the limited number of samples of East Asian genetic ancestry (*n* = 19).

The most common loss-of-function allele observed in the cohort was *3A with an overall frequency of 3.2%. The frequency of the TPMT*1/*3A diplotype was highest among individuals of Admixed American ancestry (7.5%). This diplotype was also prevalent in individuals of European ancestry at a frequency of 7.1% (Figure 2). These findings are expected, as estimates from CPIC and recent large scale studies have reported elevated frequency of the *1/*3A diplotype in European and Latino populations.^24,39^ Three individuals of European ancestry were found to be homozygous for the *3A allele (TPMT*3A/3A; Figure 2). This diplotype has been most commonly reported in individuals of European and Latino ancestry by CPIC.^24^ Across each ancestral group in this cohort, the frequency of the *3A allele was comparable to the respective CPIC allele frequency estimate (Supplementary Figure 2).

Of the samples where genetic ancestry could be inferred, TPMT*1/*3C was most common in individuals of African genetic ancestry, with a frequency of 2.8% (Figure 2). The tabulated CPIC frequencies similarly show individuals from African American/Afro-Caribbean biogeographical regions having the highest prevalence of the *1/*3C diplotype.^24^ The *3C allele is the most common *TPMT* allele in individuals of Asian ancestry^24,39^; hence the frequency of *1/*3C in participants of South Asian ancestry (1.3%) was not surprising (Figure 2). There were not any statistically significant differences between the frequency of the *3C allele in the cohort compared with CPIC estimates (Supplementary Figure 3).

**Figure 3.**
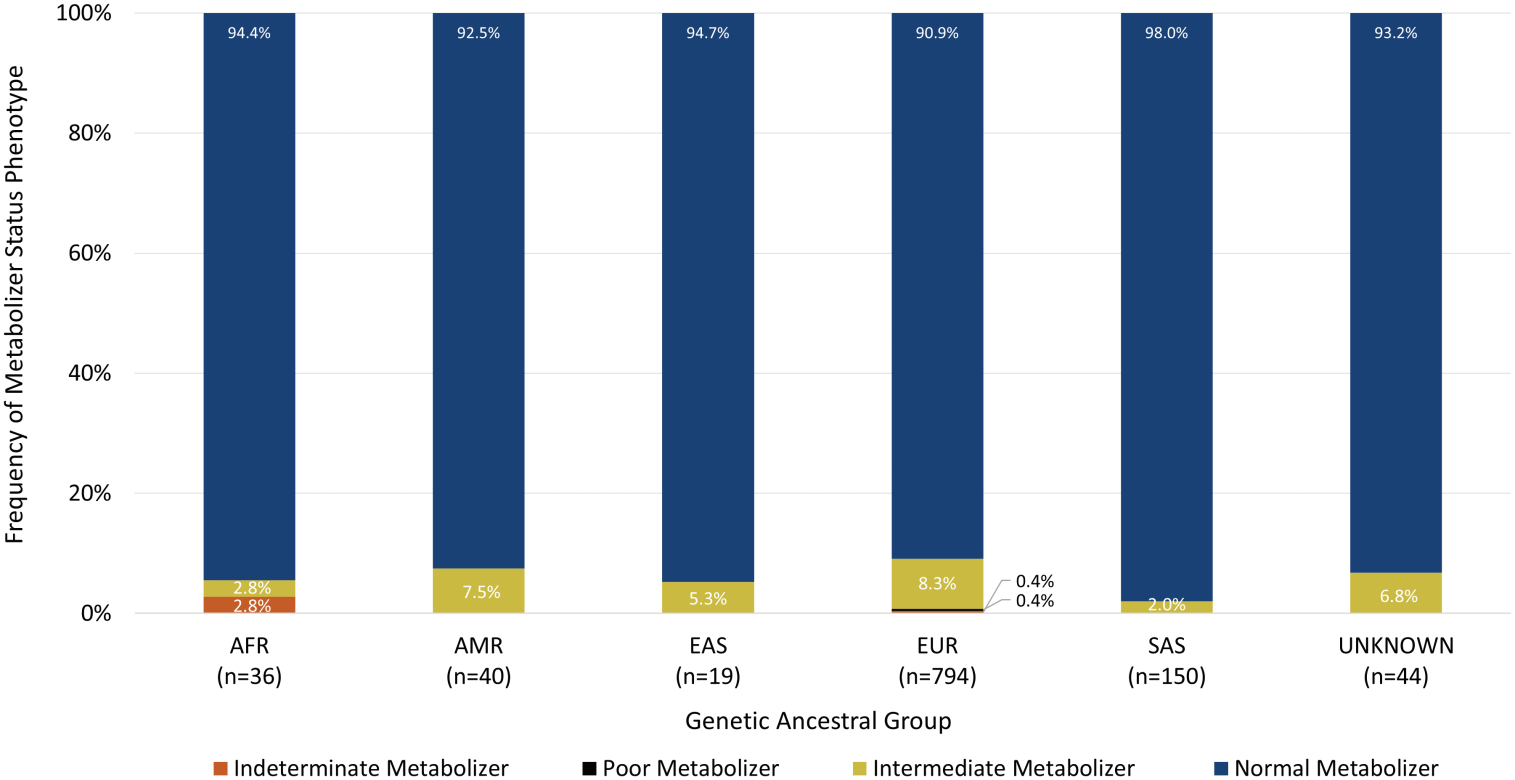
Frequency of TPMT metabolizer phenotypes observed in each genetic ancestral group within the CIDsCaNN cohort. Indeterminate metabolizer status was assigned if the participant carried an allele of uncertain function as denoted by CPIC/PharmVar. Poor metabolizer status corresponds to the detected *3A/*3A diplotype. Intermediate metabolizers had one of the following detected diplotypes: *1/*2, *1/*3A, or *1/*3C. Normal metabolizers are considered to be wild-type (*1/*1). Abbreviations: AFR, African; AMR, Admixed American; EAS, East Asian; EUR, European; SAS, South Asian. The “unknown” ancestral group corresponds to samples whereby ancestry could not be resolved by principal component analysis due to possible genetic admixture.

While the majority of the cohort was assigned NM phenotype, at least 1 clinically actionable *TPMT* result was identified in each of the 5 ancestral groups, as well as in the “unassigned” group, corresponding to participants whose genetic ancestry could not be inferred by PCA (Figure 3). The proportion of clinically actionable metabolizer status phenotypes (IM and PM) was highest in individuals of European and Admixed American genetic ancestry (8.7% and 7.5%, respectively). The differences in proportion between the genetic ancestral groups was statistically significant (*P* < .05). Of the 1083 participants, approximately 0.3% were determined to be PMs of thiopurines based on *TPMT* diplotype, all of whom were of European genetic ancestry. Based on published PGx-dosing guidelines from CPIC for the treatment of nonmalignant conditions such as IBD, these participants would be recommended to consider nonthiopurine immunosuppressant therapy. A further 7% of the cohort was determined to be IMs of thiopurines and would thus benefit from a 20% to 70% reduction of the standard starting dose as per CPIC recommendations.^19^

### *NUDT15* Genotyping

Analysis of *NUDT15* diplotypes was restricted to the loss-of-function *3 allele (Supplementary Table 1). There are 2 additional loss-of-function alleles that have been annotated by PharmVar and CPIC, *2 and *9. The *2 allele shares the defining variant c.415C > T (p.Arg139Cys) of the *3 haplotype, although the additional variant needed to uniquely differentiate *2 was imputed with insufficient quality. The variant defining *9 also did not reach the minimum threshold for imputation, and thus both alleles were excluded from analysis. Participants designated as IMs had a diplotype of NUDT15*1/*3. Participants designated as PMs had 2 copies of the loss-of-function allele, NUDT15*3/*3. No previously characterized uncertain function *NUDT15* alleles were observed in the cohort. Participants who did not carry the *3 allele were assigned wild type/normal function diplotype *1/*1.

**Figure 4.**
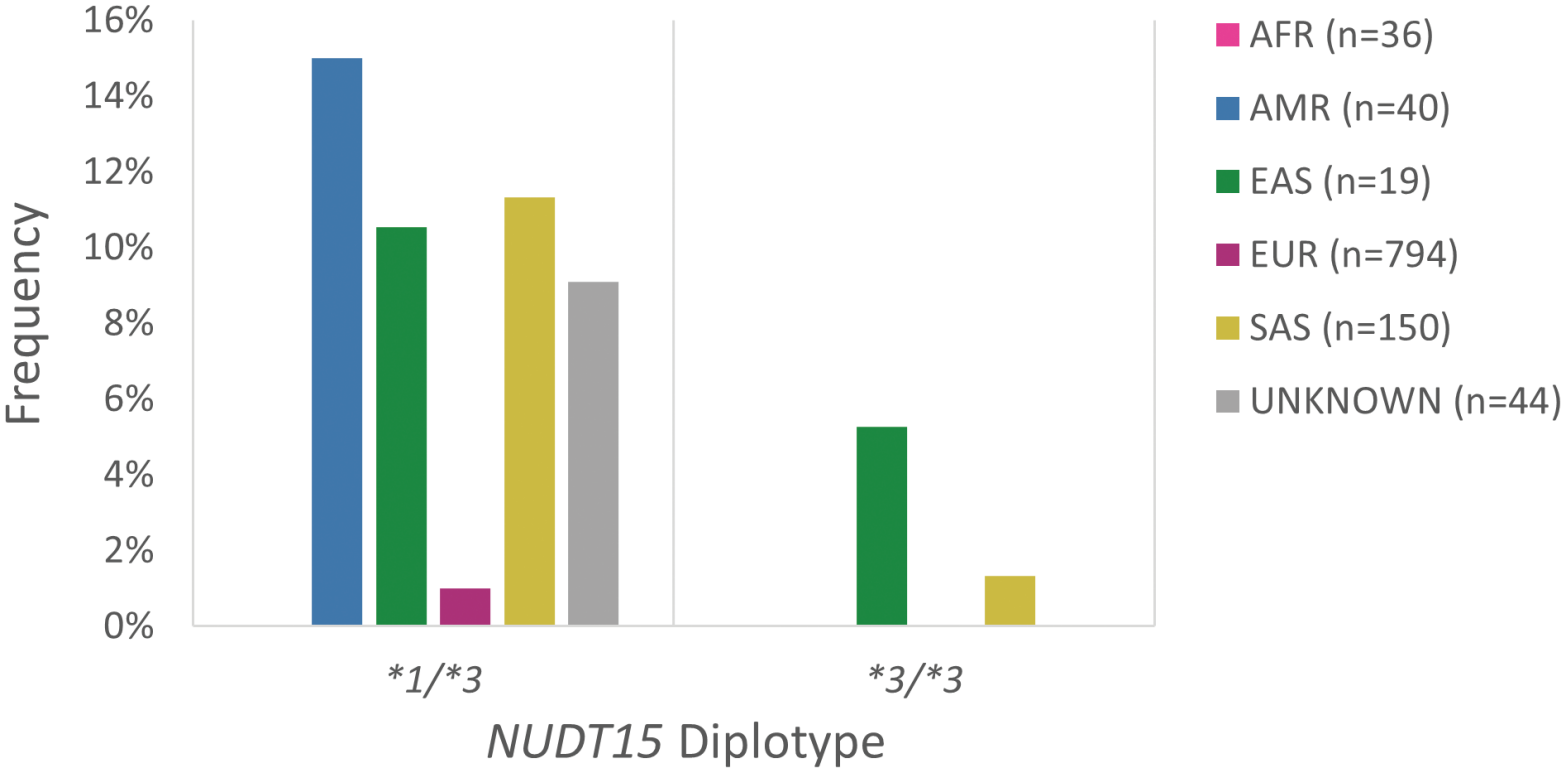
Frequency of *NUDT15* diplotypes associated with a nonstandard thiopurine dosing recommendation as per published PGx guidelines and drug label annotations, stratified by genetic ancestral group. The *1/*3 diplotype is associated with intermediate metabolizer status. The *3/*3 diplotype is associated with poor metabolizer status. Abbreviations: AFR, African; AMR, Admixed American; EAS, East Asian; EUR, European; SAS, South Asian. The “unknown” ancestral group corresponds to samples whereby ancestry could not be resolved by principal component analysis due to possible genetic admixture.

The NUDT15*1/*3 diplotype was detected in all but 1 genetic ancestral group (Figure 4). The absence of the diplotype in the African population is expected, given the relatively low frequency of the *3 allele in individuals of African ancestry within population databases, such as gnomAD. The NUDT15*1/*3 diplotype is also expected to be less common in individuals of European ancestry compared with individuals of Asian ancestry, which is reflected in this cohort (Figure 4).^27,30,40^ The diplotype frequency observed in both East and South Asian ancestry in this cohort (11% and 12% respectively) is generally consistent with estimates generated by CPIC.^29^ An interesting finding in this cohort is the frequency of the NUDT15*1/*3 diplotype (15%) in participants of Admixed American ancestry. This was the highest recorded frequency among all genetic ancestral groups. This frequency differs considerably from the CPIC tabulated diplotype frequency for individuals from Latino biogeographical regions, although this may be explained by limited number of studies that were sampled or the small sample size reported herein.^29^ Comparison of the frequency of the *3 allele between the ancestral groups comprising this cohort to that of overlapping biogeographical populations estimated by CPIC revealed a statistically significant difference (*P*  < .01) in the Admixed American and Latino groups (Supplementary Figure 4). Recent studies have demonstrated Admixed American and Latino subpopulation-specific variability in *NUDT15* that contributes to a broad range of expected allele frequencies.^41,42^ The Admixed American superpopulation in the 1KGP database is composed of individuals of Mexican, Puerto Rican, Colombian, and Peruvian ancestry, and thus is not representative of all Latino subpopulations. The populations from which the CPIC frequencies were derived do not necessarily align with the 1KGP subpopulations, which may also explain the differing frequencies.

Participants with the NUDT15*3/*3 diplotype (PM phenotype) were of East and South Asian genetic ancestry. Individuals with 2 copies of a loss-of-function allele in *NUDT15* are considered enzyme deficient. NUDT15 enzyme deficiency is more common in individuals of Asian and Latino ancestry. Approximately 2% of the East Asian population is expected to be NUDT15 deficient.^19^ The frequency of NUDT15*3/*3 in participants of East Asian genetic ancestry was notable in this cohort (5.3%) but may appear inflated due to limited sample size (*n* = 19; Figure 4).

**Figure 5.**
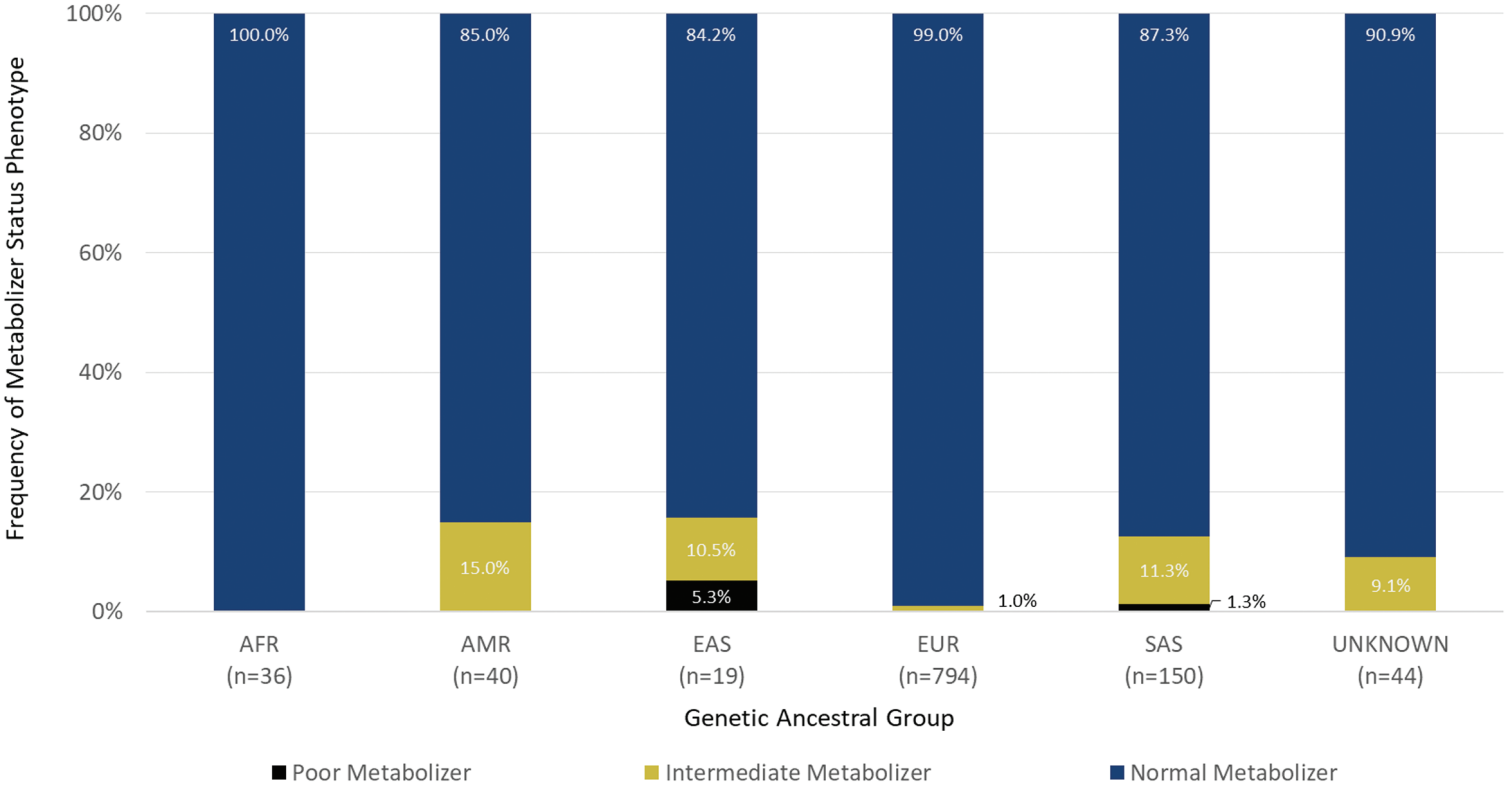
Frequency of NUDT15 metabolizer phenotypes observed in each genetic ancestral group within the CIDsCaNN cohort. Poor metabolizer status corresponds to the detected *3/*3 diplotype. Intermediate metabolizers had the *1/*3 diplotype. Normal metabolizers are considered to be wild-type (*1/*1). Abbreviations: AFR, African; AMR, Admixed American; EAS, East Asian; EUR, European; SAS, South Asian. The “unknown” ancestral group corresponds to samples whereby ancestry could not be resolved by principal component analysis due to possible genetic admixture.

Unlike the findings from the *TPMT* analysis, not all genetic ancestral groups yielded clinically actionable *NUDT15* diplotypes. All participants within the African subpopulation were assigned NM status based on the absence of the *3 allele. The proportion of clinically actionable metabolizer status phenotypes was highest in individuals of Admixed American and East Asian genetic ancestry (15% and 16% respectively), although PM status was only observed in individuals of East and South Asian ancestry. The differences in proportion between the genetic ancestral groups was statistically significant (*P* < .001). Among the 1083 participants, 0.3% were genetically PMs, and 3% were IMs of thiopurines (Figure 5). Mirroring the recommendations based on *TPMT* genotyping for nonmalignant conditions such as IBD, PMs of *NUDT15* are recommended to consider nonthiopurine immunosuppressant therapy, while IMs would benefit from a reduction of the standard starting dose.

### Summary of Findings

CPIC, and certain drug approval agencies such as the FDA, provide recommendations for both standalone genotyping of *TPMT* or *NUDT15*, as well as joint genotyping when both genes are available for testing prior to initiating thiopurine therapy. Notably, the participants who were IMs or PMs of thiopurines based on *NUDT15* diplotype were NMs based on *TPMT* diplotype. Inversely, participants with clinically actionable *TPMT* results were NMs based on *NUDT15* diplotype. Clinically actionable PGx variation in *TPMT* was more common than that of *NUDT15* (7.4% vs 3.7%; Figures 3,5). On a subpopulation basis, clinically actionable variation in *TPMT* was more commonly observed in individuals of European and African genetic ancestry whereas actionable variation in *NUDT15* was more prevalent in individuals of East Asian, South Asian and Admixed American genetic ancestry. Collectively, approximately 11% of the cohort had variation in *TPMT* or *NUDT15* that is expected to reduce enzyme activity and would warrant deviation from standard dosing of azathioprine, 6-mercaptopurine, and thioguanine based on published clinical guidelines.

## Discussion

To our knowledge, this is one of the only studies that assessed the prevalence of pharmacogenetic variation in *TPMT and NUDT15* in a multicenter pediatric IBD cohort. Increasing global incidence of IBD necessitates consideration of PGx variation that is representative of diverse populations. Canada hosts a diverse population that is hence particularly well-suited to study interpopulation PGx variability. Array based genotyping offers the ability to interrogate many variants of clinical interest. Although several previously characterized loss-of-function alleles in both *TPMT* and *NUDT15* were not captured by the Illumina GSA panel, nor imputed with sufficient quality, the loss-of-function alleles reported in this study comprise the key alleles recommended by the Association for Molecular Pathology for clinical testing.^43^ The allele frequencies observed in the CIDsCaNN cohort largely align with population data previously annotated by CPIC, apart from TPMT*2 in the East Asian group and NUDT15*3 in the Admixed American group. Comparison of the detected population-specific allele frequencies to the corresponding estimates from CPIC serves as a point of reference to visualize how the frequencies reported herein compare to previous global estimates. With that said, the allele frequency estimates aggregated by CPIC largely come from publications that have used self-reported race and ethnicity rather than genetic ancestry. This frequency data is extracted from a mixture of studies, some comprised solely of healthy controls, and some with patients who were exposed to thiopurines.^24^ It is important to note that the last major update of the CPIC *TPMT* and *NUDT15* frequency tables was in 2018, therefore these estimates are outdated and limited by sample size and subpopulation sampling. This is exemplified by a recent meta-analysis of *NUDT15* variation in diverse populations whereby the cumulative frequency of the *1/*3 diplotype was 18% and 20% in patients of Chinese and Japanese ancestry, respectively.^27^ The same diplotype is annotated by CPIC as having a frequency of 11% in individuals of East Asian biogeographical origin.^29^ The limited sample size of individuals of East Asian, African, and Admixed American genetic ancestry in this cohort may contribute to an inflated estimation of the frequency of loss-of-function alleles. It is also possible that the proportion of NMs is inflated given the inability to detect all known PGx variation in both *TPMT and NUDT15,* leading to a default assignment of wild-type diplotype (*1/*1). The undetectable alleles account for 0.11% to 3.3% and 0.30% to 2.6% of annotated PGx variation in *TPMT* and *NUDT15*, respectively, as per prior population-specific estimates. Another limitation inherent in this study is the inference of genetic ancestry by PCA for populations that are underrepresented or absent from the 1KGP cohort, including North African and Middle Eastern ancestries. Participants who reported these backgrounds predominantly clustered genetically with European ancestry.

The distribution of PGx variation and corresponding metabolizer status was overall consistent with prior global estimates. As expected, intermediate and poor metabolizer status associated with *TPMT* diplotype was more prevalent in participants of European and African ancestry.^19^ Notably, clinically actionable PGx variation in *TPMT* was observed in each genetic ancestral group, ranging from 2% of participants of South Asian ancestry to 8.7% of participants of European ancestry. Variation in *TPMT* has been previously associated with adverse reactions to thiopurines in IBD cohorts.^26^ The detection of TPMT*2 in individuals of Asian ancestry, particularly East Asian, is a relatively uncommon finding.^24^ Although the allele frequency may be inflated due to the limited sample size of individuals of East Asian genetic ancestry, detection of such PGx variation in *TPMT* supports the need to further characterize allele frequency and associated clinical outcomes in patients of differing ancestral backgrounds.

As anticipated, variation in *NUDT15* conferred a higher proportion of IMs and PMs in participants of East Asian, South Asian, and Admixed American ancestry. Actionable PGx variation was observed in each of the ancestral groups, barring African. The proportion of clinically actionable *NUDT15* variation ranged from 1% (European ancestry) to 15.8% (East Asian ancestry). Detection of NUDT15*3 at a frequency of 1.0% in participants of European genetic ancestry is a notable finding. Surmounting evidence supports the influence of *NUDT15* variation on the development of toxicity in individuals of European ancestry, including TIM in patients with IBD who have been exposed to AZA and 6-MP.^23,44^ Additionally, the combination of loss-of-function alleles in both *TPMT* and *NUDT15* has been associated with an increased probability of developing adverse reactions to thiopurines.^23^ Thus, there is a growing body of evidence supporting the merit of testing *NUDT15* in individuals of European ancestry, in addition to Asian and Admixed American ancestry.

In this cohort, none of the participants simultaneously harbored actionable PGx variation in both genes, pointing to the utility of joint genotyping to identify individuals at risk of thiopurine-induced adverse reactions. Furthermore, the proportion of PMs with inherited deficiency in *TPMT* and *NUDT15* was equal (0.3%). Recommendations from consortia such as CPIC, DPWG, and existing product monographs issued by the FDA, EMA, as well as Swissmedic, highlight the importance of genotyping both *TPMT* and *NUDT15.* These recommendations in conjunction with updated allele frequency information should be considered in clinical practice, particularly in settings that serve diverse populations. Findings from a preliminary study across 30 Dutch hospitals suggested that the financial impact of *TPMT* genotyping in the context of IBD would be neutral for the healthcare system.^45^ Nevertheless, further large scale and long-term studies are needed to shed more light on the economic value of PGx-guided thiopurine dosing. It will also be important to evaluate the financial impact of preemptive *NUDT15* genotyping, as it relates to the mitigation of adverse reactions in patients on thiopurine therapy.

Continued use of thiopurines in the care of pediatric IBD patients warrants a proactive approach to pharmacotherapy, given the risk of severe side effects such as myelosuppression. Pharmacogenetic testing should be leveraged to identify individuals who would benefit from a reduced starting dose or alternative therapy in advance of thiopurine treatment. The distribution of pharmacogenetic variation observed in this diverse cohort of Canadian pediatric IBD patients supports the utility of both *TPMT* and *NUDT15* genotyping to provide the best care possible for patients in advance of thiopurine treatment.

## Supplementary Data

Supplementary data is available at *Inflammatory Bowel Diseases* online.

izae109_suppl_Supplementary

## Data Availability

Additional de-identified data may be shared upon reasonable request and with fulfilled legal requirements (approval from all ethics committees and data transfer agreements).
