# Supplementary material for: Landscape of TPMT and NUDT15 Pharmacogenetic Variation in a Cohort of Canadian Pediatric Inflammatory Bowel Disease Patients
Source: Inflamm Bowel Dis. 2024 May 24;30(12):2418–27. doi: 10.1093/ibd/izae109 (PMC11630297; doi:10.1093/ibd/izae109)
Supplement: izae109_suppl_Supplementary [file izae109_suppl_supplementary.docx]

**Supplementary Table 1.** Star Allele Descriptions

| Star Allele | HGVS Reference Sequences for Defining Variant(s) |
| --- | --- |
| *TPMT*2* | NM_000367.5:c.238G>C (p.Ala80Pro) |
| *TPMT*3A* | NM_000367.5:c.460G>A (p.Ala154Thr)  NM_000367.5:c.719A>G (p.Tyr240Cys) |
| *TPMT*3B* | NM_000367.5:c.460G>A (p.Ala154Thr) |
| *TPMT*3C* | NM_000367.5:c.719A>G (p.Tyr240Cys) |
| *NUDT15*3* | NM_018283.4:c.415C>T (p.Arg139Cys) |

Abbreviations: HGVS, Human Genome Variation Society.

A summary of the variants defining each of the loss-of-function star alleles detected in the CIDsCaNN cohort.

**Supplementary Figure 1.** Frequency of *TPMT*2* in the CIDsCaNN Cohort Compared to CPIC Estimates

Frequency of the *TPMT*2* allele observed in the CIDsCaNN cohort as compared to estimates tabulated by CPIC, stratified by genetic ancestral group. A χ2 test was used to evaluate differences in frequency; a p-value of <0.05 was considered statistically significant. In individuals of East Asian ancestry, the frequency of *TPMT*2* was significantly higher (p-value <0.001***) in the study cohort compared to global CPIC estimates.

**Supplementary Figure 2.** Frequency of *TPMT*3A* in the CIDsCaNN Cohort Compared to CPIC Estimates

Frequency of the *TPMT*3A* allele observed in the CIDsCaNN cohort as compared to estimates tabulated by CPIC, stratified by genetic ancestral group. A χ2 test was used to evaluate differences in frequency; a p-value of <0.05 was considered statistically significant. No statistically significant differences were identified.

**Supplementary Figure 3.** Frequency of *TPMT*3C* in the CIDsCaNN Cohort Compared to CPIC Estimates

Frequency of the *TPMT*3C* allele observed in the CIDsCaNN cohort as compared to estimates tabulated by CPIC, stratified by genetic ancestral group. A χ2 test was used to evaluate differences in frequency; a p-value of <0.05 was considered statistically significant. No statistically significant differences were identified.

**Supplementary Figure 4.** Frequency of *NUDT15*3* in the CIDsCaNN Cohort Compared to CPIC Estimates

Frequency of the *NUDT15*3* allele observed in the CIDsCaNN cohort as compared to estimates tabulated by CPIC, stratified by genetic ancestral group. A χ2 test was used to evaluate differences in frequency; a p-value of <0.05 was considered statistically significant. In individuals of Admixed American ancestry, the frequency of *NUDT15*3* was significantly higher (p-value <0.01**) in the study cohort compared to global CPIC estimates. CPIC does not include an estimate of *NUDT15*3* allele frequency for individuals of African Ancestry, therefore this population was excluded.
